# Exploring Duckweed Diversity at the Dawn of Its Cultivation Era: The Invaluable Legacy of the Landolt Collection

**DOI:** 10.3390/plants15030345

**Published:** 2026-01-23

**Authors:** Laura Morello, Yuri Lee, Luca Braglia

**Affiliations:** Institute of Agricultural Biology and Biotechnology, National Research Council (CNR-IBBA), Via Alfonso Corti 12, 20133 Milano, Italy; yulee.lee@ibba.cnr.it (Y.L.); luca.braglia@cnr.it (L.B.)

**Keywords:** duckweed, Lemnaceae taxonomy, new crops, germplasm collections, genetic diversity, domestication

## Abstract

The aquatic plant family Lemnaceae, commonly called duckweed or water lentil, has attracted increasing interest in the scientific literature over the past two decades. It holds extraordinary potential as a new crop due to its multiple applications: as an alternative protein source for feed and food production, as a starch producer for renewable biofuel, and for its capacity to provide valuable ecosystem services. Its high biomass productivity, ability to thrive under a wide range of environmental conditions, lack of requirement for arable land, and aptitude for nutrient recycling from wastewater align with the criteria for future sustainable crops. The Lemnaceae is a small plant family comprising a still uncertain number of species and hybrids with largely unexplored genetic diversity, owing to its taxonomic complexity. We focus on critical aspects that must be addressed to establish duckweed as a viable crop: the availability and accessibility of genomic resources to understand the genetic basis of key agronomic traits; the development of protocols for flower induction and crossing; and the establishment of effective methods for genetic transformation and plant regeneration, all aimed at enabling selection and breeding strategies. We highlight the importance of duckweed germplasm collections, including accessions from a wide geographic and ecological range, as essential resources for addressing duckweed diversity and supporting both fundamental research and agronomic applications.

## 1. Introduction

Duckweed, the common name for the Lemnaceae (Martinov) family, is a cosmopolitan aquatic monocot inhabiting freshwater systems worldwide. Under favorable, eutrophic conditions, most species can rapidly cover the surface of entire water bodies within days through asexual propagation. Nevertheless, given the considerable economic potential of these plants, this vigorous growth capacity can be viewed as a favorable trait for productivity. Indeed, duckweed is welcomed as a potential new crop, intended as a plant that can be grown and harvested for profit or subsistence. Owing to its optimal amino acid profile and high vitamin content, duckweed is suitable as an alternative protein source for human and animal nutrition [1,2,3]. It can also be exploited for bioenergy production by redirecting its metabolism toward starch accumulation through nitrogen starvation [4].

Duckweed can be cultivated in shallow water with minimal nutrient input, across various scales and systems (closed or open, indoor or outdoor), depending on the intended application (e.g., feed or food production). Small-scale cultivation facilities have even been proposed as bioregenerative life-support systems for space missions [5]. Its intrinsically high growth rate supports the first essential crop trait—high biomass yield—which can be coupled with nutrient recycling from agro-industrial effluents. Moreover, duckweed cultivation requires low water input and no arable land, thereby minimizing environmental impact, provided that the potential ecological impact is carefully evaluated.

Along with other Freshwater Aquatic Macrophytes (FAMs) commonly grown in South and Southeast Asia, duckweed has traditionally served as food—especially in Thailand—and as feed for fish and poultry in various regions [6]. Increasing water pollution associated with intensive livestock farming and industrial wastewater discharge has raised significant safety concerns, substantially limiting the use of duckweed for direct human consumption. Nevertheless, duckweed cultivation is a great promise for tropical and temperate developing regions, where it can represent both a financially viable and environmentally sustainable production system. The establishment of controlled environment farming practices represents the first step toward duckweed domestication, the process of artificially selecting plants to increase their suitability for human needs.

The era of commercial duckweed production has already begun, with a growing number of companies in several countries marketing edible products—primarily as high-value “superfood” items enriched in vitamin B12 and flavonoids, or as food supplements in the form of dried protein concentrates [7]. However, cultivation remains restricted to only a few species within the genera *Lemna* L. and *Wolffia* Horkel ex Schleid., often with limited or no genotype-based selection. Several technical challenges still constrain the economic viability of large-scale production for feed or bioenergy purposes, including difficulties in scaling up cultivation, developing efficient harvesting technologies, controlling algal competition, and reducing drying and downstream processing costs. This perspective paper summarizes recent advances in duckweed biology in view of its possible domestication. We discuss challenges and opportunities and identify the main knowledge gaps that need to be filled in order to assist the development of new crop species from wild ancestors. For a more detailed treatment of duckweed ecology, physiology, biogeography, and phytoremediation applications, we refer to the following references [8,9,10,11,12].

Critical aspects for domestication include taxonomic complexity, impairing species delimitation; limited availability of genomic resources; and limited knowledge of sexual propagation and mating strategies. Ongoing and future studies are greatly aided by the availability of a remarkable germplasm resource, a legacy of Prof. Elias Landolt, the importance of which is also described, along with the need to preserve and valorize it.

## 2. Turning a Weed into a Crop

The growing world population, extreme seasonal events caused by climate change, and increasing loss of arable land represent growing threats to agriculture and global food security. Among others, the development of new crops has emerged as a promising approach, opening a new era of domestication [13,14]. Humanity still relies on a few highly successful crop species, independently domesticated on different continents starting from roughly 10,000 years ago, giving rise to the thousands of extant cultivars that humans have adapted to a wide range of environments through selection and breeding practices.

The need to broaden this restricted crop palette is now widely recognized. Most current efforts focus on wild relatives of existing crops, aiming to select elite foundation material—often characterized by enhanced resistance to biotic and abiotic stresses—for the introgression of domestication-related traits through modern genetic and breeding tools, in order to meet emerging agricultural challenges [15].

By contrast, de novo domestication of entirely new crop species, including those unrelated to traditional crops such as agave, seaweeds, and microalgae, is still in its infancy. This approach faces substantial challenges, including the lack of established cultivation and harvesting practices as well as the scarcity of essential genetic and phenotypic data, such as high-quality reference genomes [16]. Nevertheless, these limitations can be overcome by harnessing recent technological advances that provide unprecedented access to genomic information, thereby accelerating the identification of key desirable traits for genotype selection and breeding programs.

At the same time, the development of new crops offers novel opportunities, for example, the possibility of designing innovative cultivation systems, utilizing marginal or degraded lands, or, in the case of aquatic species, recovering nutrients from agricultural or agro-industrial wastewater. The most desirable new crops should combine multipurpose use with low-input cultivation requirements. In addition, they may contribute to the restoration of degraded and threatened ecosystems. Finally, careful attention should be given to maintaining intraspecific diversity, including cryptic genetic variation that has often been lost in traditional crops through the classical domestication bottleneck [13,17].

Duckweed fulfills many of these requirements and represents a promising plant as a new crop [2,3]. All genera within the Lemnaceae share several key traits: a floating aquatic lifestyle, small size, rapid asexual propagation [8,9], high protein content in their whole edible plant bodies, and a proximate composition comparable across species [2,7]. While these traits are differently expressed across the wide ecological diversity of duckweed species, they collectively enable natural adaptation to a broad range of environments and climates, explaining the global colonization of freshwater habitats [8].

## 3. Taxonomic Complexity: Why Taxonomy Matters?

Taxonomy, the ranked system of categorizing and unequivocally naming living organisms, introduced with the binomial classification by Linnaeus in the 17th century, is not limited to satisfying the human attitude (and need) to name and classify natural objects. Species categorization, although still debated and not unequivocal, is at the basis of systematic studies, allowing for the reconstruction of evolutionary trees in all kingdoms. In principle, individuals belonging to the same species are expected to be interfertile and give fertile progeny, have very similar morphological (structural), physiological (metabolic), and genetic features, and share a common set of allelic variants.

Taxonomy is fundamental to understanding biodiversity and is a cornerstone for the conservation and sustainable management of plant resources, including food crops. It helps researchers identify species evolution and relationships, enabling the selection of appropriate crop plant materials for breeding programs and other studies.

The taxonomic complexity of the Lemnaceae and the difficulty in assigning wild specimens to the appropriate species can compromise the successful domestication and breeding of plants belonging to different taxa if their systematic position and relationships are not well defined. Last but not least, when dealing with food plants, legal issues may arise as only certain species may be allowed on the market by local regulations, making it important to have unequivocal identification of each cultivated duckweed clone at the species level. In the European Union, only four species, namely *Lemna minor* L., *L*. *gibba* L., *Wolffia arrhiza* (L.) Horkel ex Wimm., and *W. globosa* (Roxb.) Hartog and Plas have been approved as human food by the relevant authorities [18,19]. Until a substantial equivalence of other duckweed species is demonstrated, there is a need for producers to be able to verify species identity. In this regard, it has to be noted that a clear delimitation of the species *W*. *globosa* is still lacking, as this species cannot be clearly separated from the closely related Asian species *W*. *neglecta* Landolt by any molecular marker [20]. We provide here a summary of the recent taxonomic progress.

## 4. Duckweed Taxonomy in Brief, from Linnaeus to Landolt and Beyond

Although the common name “duckweed” is often used in the singular, it does not refer to a single species or even a single genus, but rather to an entire, albeit small, plant family, the Lemnaceae. Despite molecular phylogenetic studies placing the taxon as monophyletic with the ancient monocot family Araceae Juss., thereby downgrading it to a subfamily (Lemnoideae Engler), these entirely aquatic plants separated early from the Araceae core group and retain unique structural and physiological features that justify their consideration as a distinct family [21]. We therefore prefer to follow the original classification.

The Lemnaceae family comprises several species in five genera (Table 1). Linnaeus named the first five species, only those present at his time in Europe, under the genus *Lemna*. Descriptions of many further species by other botanists followed over time. Elias Landolt (1926–2013), the most eminent scholar of the Lemnaceae in the last century and author of a two-volume monograph on this plant family [8,9], added the last eleven new species to the list [9,22,23,24], two of which, *L*. *ecuadoriensis* Landolt and *L*. *yungensis* Landolt, were later synonymized with other species [25,26,27]. As the taxonomy of the Lemnaceae has been extensively reviewed in recent years [25,28,29], we limit our attention to the most recent changes. Thanks to Landolt’s extensive taxonomic work and his collaboration with other taxonomists who introduced him to the use of molecular markers, at the time of his death, the number of accepted species was 37, classified into five genera: *Landoltia* Les and Crawford, *Lemna* L., *Spirodela* Schleid., *Wolffia* Horkel ex Schleid., and *Wolffiella* Hegelm.

If molecular taxonomy is now recognized as a valid support to morphological classification, this is particularly true for the Lemnaceae, where morphology is extremely simplified. The reduction or loss of typical organs, such as roots and leaves, as an adaptation to a floating aquatic lifestyle, and the rarity of flowering owing to the prevalence of clonal propagation, brought about the loss of many taxonomically useful characters. Large genetic variation and phenotypic plasticity, the ability of genetically identical individuals to express different phenotypes in response to changes in abiotic or biotic conditions, also contribute to intraspecific variation, blurring species boundaries in some cases [8]. The advent of molecular taxonomy provided the opportunity to integrate the limits of morphology-based taxonomy, supporting or refuting previous assessments. Starting from biochemical markers such as flavonoids and isozymes, and extending to multiple DNA markers (fragment markers such as AFLP, ITS, SSR, as well as sequence markers, such as rDNA ITS and ETS, and several plastid regions) multiple attempts have been made during the last decades to provide support for accepted taxonomy. The integration of nuclear (ribosomal) and plastid markers led to the phylogenetic reconstruction of the Lemnaceae [30] and DNA barcoding has been recognized as the gold standard for duckweed species assignment, although, as it occurred in many other plant species, the more suitable plastid markers may differ across genera and at least two barcoding sequences are needed to unequivocally define a species [31,32].

Despite this progress, some species of the Lemnaceae could not be distinguished, forming heterogeneous phylogenetic clades whenever a quite large number of accessions have been investigated by any marker, leading to the conclusion that misclassifications or mislabeling of clones may have occurred in collections. As sometimes hypothesized, and later recognized, this was not the only problem, because frequent interspecific hybridization also plays a confounding role.

Some important updates have been made to the taxonomy of the genus *Lemna* in the last few years, which are summarized below, leading to the updated list of species and hybrids reported in Table 1:

• *Lemna valdiviana* Phil. and *L. yungensis* were put in synonymy—based on the absence of any genetic, biochemical, and morphological evidence for a separation of these two species [26]. As *L. yungensis* was found exclusively on the wet rocks behind waterfalls in the Andean Yungas in Bolivia, the observed morphological difference was likely due to different ecological adaptations.
• *Lemna × japonica* Landolt (pro sp.) was recognized as a hybrid taxon (nothospecies)—The species was described by Landolt in 1980 as distinct from *L*. *minor*, and geographically restricted to warmer regions of Far East Asia, such as southern Japan and Korea. While testing a molecular fingerprinting technique [33], Tubulin-Based Polymorphism (TBP), for its ability to provide species-specific profiles of duckweed at the species level [34], it was observed that *L*. *japonica* accessions and some *L*. *minor* clones showed overlapping profiles between those of most *L. minor* (M) and *L*. *turionifera* Landolt (T) accessions, providing evidence that interspecific hybridization have occurred. Whole genome sequencing (WGS) of the putative *L*. *minor* clone 8627, in combination with cytogenetic approaches, confirmed the hypothesis and showed that the hybrid includes allo-diploid (homoploid) and, mostly, allo-triploid cytotypes, with different combinations of the parental subgenomes (MT, MTT, MMT), but always having *L*. *minor* as the maternal parent [35,36]. *Lemna* × *japonica* has great ecological success, showing a distribution far larger than previously thought, spanning from East Asia to all of Europe. Recent field studies across Russia suggested that the hybrid occurs East to West and often coexists with one of its parents with an intermediate ability to tolerate the continental climate, whose ranges are separated from West (*L*. *minor*) to East (*L*. *turionifera*) by the Urals, and coincide with an eastward increase in continental climate conditions [37,38]. The ability to form turions, compact, starch-filled fronds surviving low temperatures, is not uniformly present in different hybrid populations and could be the key trait determining differential success across climatic regions. Further investigations of natural populations reported a wide presence of *L*. × *japonica* in Switzerland [39] and the UK [40], often in combination with *L*. *minor*, far from the natural range of *L*. *turionifera*, which is scarcely present. This suggests colonization from Asia. Not known is the reason for the apparent absence of *L*. × *japonica* from North America, where both parent species, *L*. *minor* and *L*. *turionifera,* commonly co-occur, apparently without hybridization, according to population genetic structure analysis using GBS data [41].
• A second cryptic *Lemna* hybrid, *L*. × *mediterranea* Braglia and Morello, was also identified during the genetic characterization of clones from the original Landolt collection, and derives from a cross between *L*. *minor* and *L*. *gibba* [34]. According to available specimens, its geographic distribution seems to be limited to Europe, with a possible origin in the Mediterranean basin [42]. In agreement, wild populations were found in Southern Italy [43], in the region where *L*. *symmeter* Giuga was described as a species different from the sympatric *L. gibba* [44]. A further putative triploid clone, MJ201, has been recently reported from Ireland [45]. The hybrid shows a similar chromosome pattern as *L*. × *japonica*, including allodiploid and triploid hybrids, and no tetraploid cytotypes, to date.
• A further *Lemna* hybrid is *L*. × *aoukikusa* Beppu and Murata (pro sp.) [46], which was already described as a distinct species, as it occurred with *L. symmeter*. The species was described as close to—but distinct from—*L. aequinoctialis*, but was not accepted by Landolt. The existence of morphological variants in *L. aequinoctialis*, supporting a separate taxon, was recently confirmed by Lee et al. [47], who studied field samples from Japan and Korea characterized by plastidic barcoding. A combination of molecular markers and genome in situ hybridization (GISH) by Stepanenko et al. [48] provided evidence that *L. aoukikusa* is an old, interspecific hybrid of *L*. *aequinoctialis* Welw. with the closely related *L. perpusilla* Torr. Contrary to the *L. minor* hybrids, *L*. × *aoukikusa* is so far represented only by tetraploid clones [48,49].

**Figure 1:**
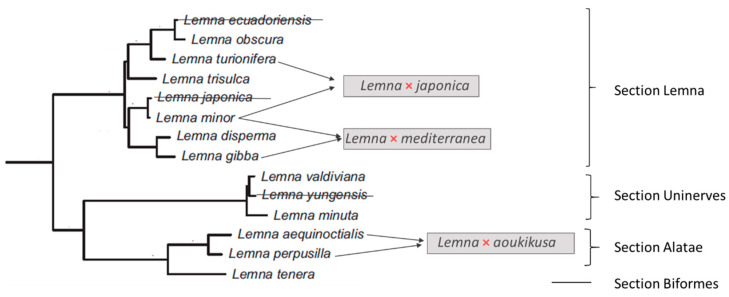
Phylogeny of the genus *Lemna*, derived from combined chloroplast and nuclear ribosomal DNA data, modified from [30], according to the last taxonomic changes. Strikethroughs indicate the two species, *L. ecuadoriensis* and *L. yungensis*, which are now considered synonyms of *L. obscura* and *L. valdiviana*, respectively, as well as *L*. *japonica*, whose phylogenetic position in the tree is revised based on its hybrid status. The number of species is reduced to 11, plus three interspecific hybrids (nothospecies).

These findings also revealed a quite high number of mislabelled accessions in the stock collections, limited to a few species and mainly due to the existence of hybrids [33,34]. Out of 58 putative *L*. *minor* clones in the RDCS, 23 (39%) turned out to be *L.* × *japonica* hybrids, most of which were triploid [36]. The accepted taxonomy of the genus *Lemna* was then mostly confirmed, with the addition of three cryptic nothospecies. In accordance, the taxonomic relationships should be revised as shown in Figure 1. Although deeper investigations in the *Lemna* section *Uninerves* may deserve further discovery of hybrids and polyploids, we can consider that little changes are expected in the taxonomy of this genus.

The same cannot be said for the other genera, *Wolffia* and *Wolffiella*, not yet deeply investigated using integrated methods, and which may conceal further unknown species or hybrids.

In conclusion, the number of species within this cosmopolitan family cannot be considered entirely defined due to its emerging taxonomic complexity. Complex patterns of hybrids and polyploids seem to shape the duckweed family, calling for the use of broader taxonomic categories such as species complexes, whose members cannot be identified without detailed molecular analysis. Species complexes usually include direct hybrids as well as backcrosses and different degrees of introgression. This does not seem to be the case with *L. minor* hybrids, which are sterile based on physiological and genetic evidence, but may occur in the *L. aequinoctialis* complex, where fertile tetraploids can backcross to the parent species [49].

Different ploidy levels within the same species, already reported since Landolt’s early studies on karyotypes, are frequent in hybrids, further increasing the phenotypic variation. These findings have great potential in view of duckweed domestication as both hybrids and polyploid plants often benefit from larger size, heterosis, genome buffering, and rapid adaptation to climate change compared to their diploid parents [15].

## 5. Genomic Resources

The Lemnaceae are characterized by a typical small size (usually 0.3–9 mm) and a simple plant body—one to a few cohering thallus-like structures conventionally called fronds, with root(s) present or absent (Landolt, 1986 [8]). These minimalist plants have attracted considerable interest due to their unique anatomy and lifestyle, which make them valuable model systems in plant biology [10,11]. This interest has fuelled a rapid expansion of duckweed genomic resources over the past decade. Since the publication of the first genome of *Spirodela polyrhiza* (L.) Schleid. more than ten years ago [50], major efforts have been devoted to expanding the number and quality of available genomes. At present, whole-genome sequences are available for nine species across four genera, though with varying levels of assembly resolution and annotation [35,51]. Additional sequencing projects are underway in several laboratories, and transcriptomic and epigenomic datasets are steadily accumulating.

Despite a progressive loss of many coding genes due to a reduction in structural complexity, duckweed genome size has increased during evolution, primarily through the accumulation of transposable and repetitive elements [52,53]. Sequencing projects started from species with the smallest genomes, 150–480 Mbp large, but the next challenge is represented by species with larger and more complex genomes, particularly those of the genus *Wolffia*, the most interesting for cultivation, having a haploid genome size up to 1800 Mbp.

The only sequenced *Wolffia* genome is that of *W. australiana* (Benth.) Hartog and Plas, which is the smallest in the genus and has high proportion of repetitive regions, consisting of both transposable elements and tandem repeats, which represent 30–39% of the genome [52,54]. Rapid advances in next-generation sequencing (NGS) technologies over the past decade strongly reduced the time and cost of the “wet part” of genome sequencing, enabling the generation of vast genomic datasets. Third-generation sequencing (TGS) platforms now provide long reads—up to 200 Kb—that greatly facilitate complex genome assembly. However, resolving the complexity of large genomes leading to the de novo assembly of an accurate reference genome still represents a challenge and requires advanced computational tools and bioinformatics expertise to handle and analyze large datasets, particularly for wild or less-studied species.

Beyond their fundamental role in understanding genome architecture, evolution, and biology, these sequencing efforts provide a strategic foundation for applied genomic research. To this purpose, it is urgent to create highly curated and annotated reference genomes, following priorities dictated by the relevance of the species with the greatest cultivation potential. Genome resequencing of many different ecotypes of each species of interest will enable the integration of genomic and phenotypic data for the identification of key agronomical traits through Genome-Wide Association Studies (GWAS). The typical, well-studied, domestication traits of food crops, e.g., reduced seed dormancy, larger and more numerous fruits or seeds, faster plant growth, and seed retention in panicles, do not apply to duckweed, making it very different from any common crop plant.

For larger genomes, alternative genome-wide genotyping methods, leveraging reduced representation libraries, remain invaluable to find genetic variations linked to traits. Techniques such as genotyping-by-sequencing (GBS), Restriction Site-Associated DNA sequencing (RAD-seq), and multiplexed ISSR genotyping-by-sequencing (MIG-seq) can efficiently detect single-nucleotide polymorphisms (SNPs) without the need for a complete reference genome. These approaches would be particularly suited for genetic mapping and marker-assisted breeding in duckweeds.

A fundamental prerequisite for all genomic studies, however, is the availability of accurately identified genetic resources—a task not to be taken for granted in duckweed, given the taxonomic complexity and frequent misidentification of clones.

## 6. Facultative Sexuality and Breeding Challenges

Since the dawn of agriculture, crop species have been selected by early farmers through the propagation or crossing of plants exhibiting desirable traits. Unlike conventional crops, duckweed species propagate asexually under favorable conditions, producing new clonal propagules from meristematic pouches, and are thought to rely on sexual reproduction mainly under stress, although conditions controlling flowering in nature are poorly understood [8]. Facultative sexuality, characterized by the rarity of flowering in nature, limits the use of classical breeding methods based on crossing selected parents to combine beneficial traits, followed by self-fertilization or backcrossing to fix introgressed traits.

However, floral induction in vitro through hormonal treatment or photoperiod can overcome this limitation [47,55,56]. Successful flowering has been achieved in a few species, whose number is expected to increase with more systematic approaches. Seed setting remains challenging and requires optimization. Each individual plant, consisting of a single frond, produces only one flower bearing 1–4 ovules, depending on the species, thus producing only one to a few seeds. Flowers are bisexual, bearing one pistil and one or two stamens depending on the genus. Knowledge of mating systems influencing the degree of heterozygosity of populations is still limited, with reported variation among species, from complete homogamy to protogyny, and from self-fertility to self-incompatibility [46,47,49]. In some cases, multiple mating strategies occur within the same species. Understanding these traits is essential for designing effective breeding strategies and selecting suitable genotypes as scaffolds for trait introgression.

Direct manual crossing is highly challenging and labor intensive due to the extremely reduced flower size, limited pollen amount from each flower, and production of single or a few seeds from each plant, but it is nonetheless feasible, at least for the genus *Lemna* [49,57]. However, cross-pollination is easily obtained by gentle agitation of the liquid culture medium. In addition, knowing the natural vectors of cross-pollination, presumed to be small aquatic animals [8], may suggest the use of such pollinators to drive breeding efforts.

Low seed yield can be counterbalanced by duckweed having the shortest generation time among all angiosperms: once produced, seeds germinate in a few days, and the newly asexually generated fronds are soon able to produce new flowers and seed upon inducing flowering in vitro [49]. The whole sexual generation time can then take no more than 30–40 days in *Lemna*. In addition, fast clonal propagation allows a newly introduced trait to be stably transmitted in the descendant population in a very short time.

The discovery of natural interspecific hybrids in the *Lemna* genus further widens breeding opportunities, showing a natural way to overcome species barriers to introduce desired traits. Knowing which species are cross-fertile and whether artificial hybrids can be obtained becomes fundamental in this respect. When hybrid vigor exists, it can be rapidly fixed by clonal propagation.

Manual crossing strategies, while challenging, are viable for the three genera with the largest fronds, *Spirodela*, *Landoltia,* and *Lemna*; they seem unfeasible for *Wolffia* due to the size of the plant itself, reaching 0.3–0.4 mm in some species. Screening and selection of best-fit genotypes depending on the application would currently be the best approach for developing elite varieties of *Wolffia*. Selection must prioritize the use of native species, at least for open cultivation, to avoid the risk of environmental dispersal of alien, potentially invasive species.

Genetic improvement can also be achieved through conventional genetic engineering technologies, including transgenesis or Assisted Evolution Techniques (TEA), such as cisgenesis and genome editing—alternative strategies more acceptable in countries, such as those belonging to the EU, which do not allow transgenic crops as food. Although transgenic plants have been obtained through *Agrobacterium*-mediated transformation [58] and genome editing has also proven successful in *L*. *aequinoctialis* [59], reports are still limited, efficiency is low, and straightforward protocols of selection and regeneration are not yet available for all species of interest. As for conventional crops, some genotypes may be more responsive than others to genetic manipulation and regeneration. Additional work needs to be performed in this direction. The development of transformation protocols could also represent an alternative option for *Wolffia*. Very little is known about the genus *Wolffiella*, which has never been tested for any application.

## 7. The Extraordinary Landolt’s Legacy: A Distributed Pan-Collection

One of the aspects that clearly emerged in all taxonomic efforts is that large numbers of clonal accessions have to be analyzed and compared in order to provide reliable data, which emphasizes the value of stock collections.

The availability of ex situ germplasm collections maintained as seed stocks of cultivated and wild plants is fundamental for the purpose of plant breeding, food security, and future needs, in addition to contributing to the storage and preservation of natural genetic diversity for research purposes.

Botanical gardens host a wide variety of wild plants from all over the world, sometimes including some duckweed species. However, cultivation in open tanks or ponds with other aquatic plants may cause the original population to be easily contaminated by individuals from the environment. The best way to preserve duckweed germplasm collections, instead, is through axenic in vitro cultivation of individual clonal lineages: a sort of Lilliputian, indoor, botanical garden in Petri dishes.

The largest ever, and most famous systematic duckweed collection worldwide was initiated in the 1950s by E. Landolt, at the University of Zurich [60]. The collection expanded gradually to include specimens from all continents, in part collected by Landolt himself during his many expeditions (Venezuela, Australia, Africa, to cite a few) and partly acquired from the many scientists who relied on his ability to correctly classify wild specimens. Each specimen was carefully identified by morphology, sterilized by bleaching, and clonally propagated in agarized medium in glass tubes. At its top size, this collection included around 2000 clones, 1000 of which were still viable at Landolt’s death [61]. This historical germplasm collection has served and continues to serve as a reference for duckweed scientists worldwide and represents a fundamental part of Landolt’s legacy, together with dozens of papers he authored on the topic [62].

Over time, a partial replicate of the collection was sent overseas to a private company in the US and is now integrated into the Rutgers Duckweed Stock Collection [63]. Further sub-collections are spread across Europe, the largest of which is at the Matthias Schleiden Institute of the University of Jena (Prof. K.-J. Appenroth). Other clones are present in collections in India, China, Japan, and Australia, and the list of these is continuously updated in the Duckweed Forum, the Newsletter of the International Steering Committee on Duckweed Research and Applications (ICDRA). In 2021, the remains of the original Landolt collection, until then privately maintained by Landolt’s former assistant Walter Lämmler, were transferred to the Institute of Agricultural Biology and Biotechnology (IBBA) in Milan, under the agreement that each clone would be genotyped using molecular markers to confirm its taxonomic assignment.

To date, approximately 1000 clonal accessions of the original living plant repository are propagated in different collections, representing a fundamental resource that should be preserved to address future challenges. Collection clone redundancy is fundamental, considering that the occasional loss of single accessions is an almost unavoidable occurrence. In addition, mislabelling of some accessions may occur over time, requiring the rescue of the original clone. An additional problem may arise from the occurrence of mutations or polyploidization upon long-term cultivation, as it has been documented in a few cases [64].

## 8. New Frontiers for Preservation: Seeds, Turions, and Meristematic Tissues

Major advantages of duckweed stock collections are the small amount of space required and mild cultivation conditions, offset by relevant challenges such as the cost and labor needed for media preparation, sterilization, and plant subculturing in axenic conditions. Laborious protocols are to be followed for the disinfection of plant material due to recurrent contamination, sometimes unavoidable due to the overgrowth of endophytic bacteria. In addition, like most germplasm collections, duckweed stock collections suffer from a scarcity of resources that should be provided at a global level to maintain such facilities. Duckweed germplasm is not stored as seed stocks because seeds are rarely produced, implying that little information about seed preservation is available. The standardization of flower induction conditions and seed production for all species will, nevertheless, open such a possibility. Promising and still poorly investigated alternatives for long-term preservation include cryopreservation [65] or the production of synthetic seeds, encapsulated meristematic tissue having the potential to regrow after storage [66]. Stress tolerance forms, such as resting fronds and turions, naturally produced by many duckweed species to survive cold or dehydration [67], may also be considered for this purpose. Effort should be put into the development, optimization, and standardization of protocols for the preservation of such forms, which may differ among genera and species. Low efficiency of preservation can be compensated for by the fast and clonal propagation of recovered plants, which preserve the original, identical genetic background.

## 9. Data Sharing and Accessibility

The original Landolt collection was accompanied by data related to each accession, such as the collection site and date, although not georeferenced, which are necessary for biogeographic and ecological studies. These data are sometimes provided by publications in which clones have been used, upon request to the curators of the collections, or through dedicated or general websites [62,68,69].

A large body of information, from physiological and genetic investigations on many collection clones, has been accumulated over time, by Landolt himself in his monograph [8,9], as well as by the many scientists to whom he provided his clones, and is available in the duckweed literature, but not easily accessible. A meta-analysis of the data related to collection clones would be a precious resource, possibly affordable with the help of Artificial Intelligence tools.

Genetic information related to plastid barcoding sequences, whole plastome sequences, and some nuclear marker genes, e.g., ribosomal spacer sequences, is mostly retrievable from GenBank [70]. Genomic, transcriptomic, and epigenomic data are also increasing and made available through different repositories after publication [71,72].

A fundamental aspect for a profitable use of genetic resources is data systematizing and sharing. The best approach would be to generate an integrated database, including available physiological, morphological, and genetic data of all clones of the Landolt collection, as well as the many clones collected and investigated in the different countries. Such integration would be invaluable both for application purposes and for basic research using duckweed as a model plant. This would need strong coordination and collaboration of scientists from different institutions worldwide, feasible only with dedicated funding [73]. This is a general problem faced by scientific collections, requiring a systematized system of archiving and sharing metadata to guarantee open access and to connect existing databases. An opportunity at the European level is represented by a nascent European Research Infrastructure, the Distributed System of Scientific Collections (DiSSCo), aimed at creating a digitally unified and harmonized system of digitized Natural Science Collections, including germplasm collections [74].

Data sharing also ensures compliance with Findable, Accessible, Interoperable, and Reusable data (FAIR) [75] principles and International treaties on plant genetic resources, the Convention on Biological Diversity, and the Nagoya Protocol about exchange and use of germplasm resources. Compliance with these treaties could be more challenging when using genetic resources from third countries for commercial purposes.

## 10. Conclusions and Future Directions

The era of duckweed cultivation has already begun, but its domestication has not. Domestication implies a long process of genotype selection and crossing to develop plants with desirable traits that ensure profitable and sustainable biomass production. To achieve this goal, a large body of information is still needed, including clarification of duckweed taxonomy and species delimitation in light of recent findings, the availability of genomic resources, and insights into sexual propagation and mating strategies. A logical starting point toward duckweed domestication is the identification of desired traits to increase the productivity of a plant (family) that is in no way comparable to any other traditional crop for its unique properties. Major targets could include a high growth rate, fast nutrient uptake, stress tolerance, and prevalence of protein or starch accumulation. The genetic bases of such traits are almost unknown, underscoring the need for extensive genome sequencing and genotype–phenotype association studies. Suitable species and genotypes for the different applications must be identified through phenotypic screening to establish targeted selection and breeding programs.

Although the foundations of genome data availability are now in place, the larger and most complex genomes of key cultivated species, *W*. *arrhiza* and *W*. *globosa,* are yet to be explored. A fundamental prerequisite for genomic and pan-genomic studies is the availability of diverse, well-characterized germplasm resources, partly available in different germplasm collections but requiring further expansion and taxonomic validation. While taxonomic identification is now almost feasible for the genus *Lemna*, using molecular methods, ambiguities remain in the genus *Wolffia,* which deserves further clarification, fundamental for the development of breeding programs and to drive sequencing priorities. Simultaneously, the development of protocols for flower induction, controlled crossing, and seed production, along with improved genetic transformation and genome-editing pipelines, will be essential for implementing breeding and molecular improvement strategies.

Future progress will depend on expanding duckweed cultivation for broader human consumption, reducing large-scale production costs for competitive feed markets, and developing locally sustainable systems for rural communities. Given the ease of environmental spreading of aquatic plants, great care must be taken to develop contained indoor cultivation systems for alien species or genetically manipulated genotypes, while outdoor cultivation facilities should be reserved for local species to avoid any potential ecological impact.

In conclusion, the full exploitation of duckweed’s potential as a multiuse crop will require a concerted and collaborative effort to deepen our understanding of its genetic, epigenetic, and ecological diversity. Only by integrating genomics, breeding, and biotechnology with physiological studies will it be possible to transform this minimalist aquatic plant into a sustainable crop for the future.

## Figures and Tables

**Table 1:** Updated list of accepted species and newly proposed nothospecies in the five genera of the Lemnaceae.

Genus	Species
*Landoltia* Les and D.J. Crawford	*L*. *punctata* (G.Mey.) Les and D.J. Crawford
*Lemna * L.	*L*. *aequinoctialis* Welw.
	*L. × aoukikusa* Beppu and Murata (pro sp.)
	*L. disperma* Hegelm.
	*L. gibba* L.
	*L. × japonica* Landolt (pro sp.)
	*L. minor* L.
	*L. × mediterranea* Braglia and Morello
	*L. minuta* Kunth
	*L. obscura* (Austin) Daubs
	*L. perpusilla* Torr.
	*L. tenera* Kurz
	*L. trisulca* L.
	*L. turionifera* Landolt
	*L. valdiviana* Phil.
*Spirodela* Schleid.	*S*. *polyrhiza* (L.) Schleid.
	*S. intermedia* W.Koch
*Wolffia* Horkel ex Schleid.	*W*. *angusta* Landolt
	*W. arrhiza* (L.) Horkel ex Wimm.
	*W. australiana* (Benth.) Hartog and Plas
	*W. borealis* (Engelm. ex Hegelm.) Landolt
	*W. brasiliensis* Wedd.
	*W. columbiana* H.Karst.
	*W. cylindracea* Hegelm.
	*W. elongata* Landolt
	*W. globosa* (Roxb.) Hartog and Plas
	*W. microscopica* (Griff.) Kurz
	*W. neglecta* Landolt
*Wolffiella* Hegelm.	*W*. *caudata* Landolt
	*W. denticulata* (Hegelm.) Hegelm.
	*W. gladiata* (Hegelm.) Hegelm.
	*W. hyalina* (Delile) Monod
	*W. lingulata* (Hegelm.) Hegelm.
	*W. neotropica* Landolt
	*W. oblonga* (Phil.) Hegelm.
	*W. repanda* (Hegelm.) Monod
	*W. rotunda* Landolt
	*W. welwitschii* (Hegelm.) Monod

## Data Availability

No new data were created or analyzed in this study.

## Abbreviations

The following abbreviations are used in this manuscript:

TEA	Assisted evolution techniques
WGS	Whole genome sequencing
RDSC	Rutgers Duckweed Stock Cooperative
AFLP	Amplified-fragment length polymorphism
TBP	Tubulin-based polymorphism
FAO	Food and Agriculture Organization
SSR	Single sequence repeats
SNP	Single-nucleotide polymorphism
GISH	Genome in situ hybridization
RAD-seq	Restriction site-associated DNA sequencing
MIG-seq	Multiplexed ISSR genotyping-by-sequencing
ITS	Internal transcribed sequences
GBS	Genotyping-by-sequencing
DiSSCo	Distributed System of Scientific Collections
TGS	Third-generation sequencing
ETS	External transcribed sequences
FAIR	Findable, Accessible, Interoperable, and Reusable
ICDRA	International Conference on Duckweed Research and Applications
